# The Evaluation of Water Sorption Effects on Surface Characteristics and Color Changes in Direct and CAD/CAM Subtractively Processed Resin Composites

**DOI:** 10.3390/ma18081812

**Published:** 2025-04-15

**Authors:** Georgiana Osiceanu, Flavia Roxana Bejan, Roxana Diana Vasiliu, Sorin Daniel Porojan, Liliana Porojan

**Affiliations:** 1 Department of Dental Prostheses Technology (Dental Technology), Center for Advanced Technologies in Dental Prosthodontics, Faculty of Dental Medicine, “Victor Babes” University of Medicine and Pharmacy Timisoara, Eftimie Murgu Sq. No. 2, 300041 Timisoara, Romaniasliliana@umft.ro (L.P.); 2 Department of Oral Rehabilitation (Dental Technology), Center for Advanced Technologies in Dental Prosthodontics, Faculty of Dental Medicine, “Victor Babes” University of Medicine and Pharmacy Timisoara, Eftimie Murgu Sq. No. 2, 300041 Timisoara, Romania

**Keywords:** water sorption, optical parameters, CAD-CAM, surface properties, optical properties

## Abstract

This study investigates the influence of water immersion on direct composite resins in comparison with subtractive processed composite resins. It evaluates changes in their optical characteristics, surface roughness, solubility, and water sorption. Four CAD/CAM materials (Vita Enamic, Brilliant, Cerasmart, and Tetric) and three direct composite resins (Gradia Direct Anterior A2, Tetric Evo Ceram A2, and Filtek Z550 A2) were examined. Direct composite samples were created using a 3D-printed mold, compressed, and photopolymerized, whereas CAD/CAM samples were sectioned and processed to the same dimensions. Seventy samples (n = 10) were prepared. After dehydration and initial weighing, samples were immersed in distilled water for 30 days, with mass changes recorded at multiple time points. Surface roughness and optical parameters were measured at three stages: post-dehydration, post-immersion, and post-reconditioning. ANOVA, unpaired and paired Student’s *t*-tests, and Pearson correlation coefficient were used to evaluate the variables. The drawn conclusion was that there were significant differences both between stages and between the materials. The most stable material in terms of water sorption was Vita Enamic. Regarding the optical parameters, Brilliant, Vita, and Cerasmart displayed higher stability, while Tetric CAD, Tetric, Gradia, and Filtek showed increased sensitivity to water immersion and re-desiccation. Gradia exhibited the highest surface roughness, while the CAD-CAM materials demonstrated the greatest stability.

## 1. Introduction

Because of their esthetic qualities and wide range of colors that fit the natural shade of the tooth, composite resins have become very popular [1,2].

A typical matrix consists of monomers (Bis-GMA, UDMA, or TEGDMA), oligomers, and initiators, filled with inorganic filler particles like silica, zirconia, or glass ceramics. Regarding the monomers used, Bis-GMA is known for having a large molecular weight, UDMA for having a low viscosity, and TEGDMA is used as a diluent to lower the viscosity of the resin matrix [3]. Higher polymerization shrinkage and greater brittleness in their mechanical properties are observed in UDMA-based composites as compared to Bis-GMA composites; this is due to the shorter molecular chains of UDMA. The hydrophobic group introduced into the monomer structure can improve the composite system of UDMA, decrease water sorption, and increase solubility resistance [4].

Research indicates that composite resins using UDMA have reduced monomer release [5]. UDMA is the most often used monomer in CAD/CAM composite blocks. Urethane dimethacrylate (UDMA) has been demonstrated to have lower water sorption and solubility than Bis-GMA, and the effect of the polymer matrix on water sorption and color stability has been well described [6].

A composite material is strengthened by incorporating fillers with distinct properties compared to the resin matrix [7].

The integration of several filler materials, including silica glass, quartz, ceramic, metal, pre-polymerized particles, and natural minerals in various forms and dimensions, can improve the mechanical properties of dental composites, resulting in reduced shrinkage volume and stress and desired flowability (or viscosity).

Despite technological advances, resin-based dental composites continue to face problems, failure rates ranging from 3% to 11%/year being reported. The main problems come from secondary cavities and fractures, either within the material itself or along its edges. The limited durability and possible health risks are caused by a number of factors, including their property to draw bacterial biofilm, stress from polymerization shrinkage, low fracture resistance, incomplete material curing, high water sorption, and even potential exposure to bisphenol-A [2].

In contrast to direct resin-based composites, CAD-CAM composite resins are processed in the laboratory based on a digital design [8]. Due to the 90% polymerization rate, mechanical properties such as flexural strength, hardness, and density are improved, along with a reduced tendency to form bacterial biofilm [9,10,11].

CAD/CAM composite blocks are classified into two main types: the first type, resin–composite blocks (RCBs), made by mixing filler particles into a monomer solution, and polymer-infiltrated ceramic network (PICN) blocks [9,12]. PICN blocks consist of a pre-sintered glass-ceramic network that is infiltrated with a monomer and polymerized [9,13].

An important consideration is the water sorption rate of resin composites, as this has a direct impact on their mechanical and physical characteristics [14].

Water sorption in dental composites largely occurs in the resin matrix, depending on the resin’s chemical composition, polymerization, and filler concentration. Water sorption is the process in which water molecules diffuse into the polymer matrix, filling the gaps between the chains, which might cause swelling and dimensional changes [15,16].

The type of dental composite and its composition influence the extent of water sorption. For example, composites with a higher filler content tend to have lower water sorption, as the volume of the resin matrix available for water sorption is lower. The type of resin monomer also plays a crucial role; due to the nature of the hydroxyl groups in the monomer structure, monomers such as Bis-GMA (bisphenol A-glycidyl methacrylate) are more susceptible to water sorption [15].

The negative effects of water sorption target both the mechanical and physical properties of resin composites. Hydrophilic materials are more vulnerable to color changes and staining, which are influenced by the amount of water absorbed [6]. It can also lead to a reduction in mechanical properties, such as strength and elastic modulus, due to plasticization of the resin matrix. In addition, water sorption can cause the release of unreacted monomers and other degradation products, potentially resulting in health risks. From a clinical perspective, these changes can lead to discoloration, a decrease in bond strength, and potential failure of the restoration over time [15].

There is a relationship between the amount of water absorbed and solubility. Absorbed water can dissolve some components, such as unreacted monomers and additives. The solubility of the polymer network depends on its crosslink density as well as the filler type. Higher crosslink density composites dissolve less, which allows them to preserve their strength and structure over time [15,16].

Through industrial curing, CAD/CAM resin composites achieve a higher crosslink density in their polymer networks and experience a reduction in pores and voids. This advantage, together with a higher proportion of filler, is expected to limit the water sorption of the materials. These advances may contribute to a low rate of chemical and mechanical deterioration over time [17,18].

Many studies have pointed out the connection between the color stability of composite materials and a variety of intrinsic and extrinsic factors [19].

Changes within the resin matrix itself, modifications at the matrix/filler interface, and chemical discoloration caused by oxidation or alterations in amine catalysts are considered intrinsic factors that contribute to color changes in composite resins [19,20,21].

The clinical performance of dental materials depends significantly on water sorption, since the resin composite water sorption has negative effects on restorative materials: it can change their color, weaken their mechanical properties, reduce wear resistance, and break down the chemical bonds at the resin–filler interface [22]. Furthermore, solubility can affect the biocompatibility of the materials, decrease their volume and strength, and lead to discoloration by disrupting the filler–resin bond within the matrix [23,24,25].

Two basic methods exist in the literature for assessing color: qualitative and quantitative. The qualitative method consists of a subjective comparison between the sample and a shade guide [19]. On the other hand, the quantitative approach—which is commonly used in dentistry to assess optical characteristics—usually consists of spectrophotometry used with the CIE L*a*b* system, a reliable and precise method [26,27].

Using a common light source, observer, and particular measurement geometry, the Commission Internationale de l’Eclairage (CIE) states that the color parameters (L*, a*, and b*) of materials can be determined by means of their reflectance or transmittance spectra. ΔE values represent color variations. Inconsistencies in the CIE Lab color space are corrected by the CIEDE2000 formula, enhancing accuracy, particularly for minor color changes [28,29].

Thus, this study aimed to investigate the effects of water immersion on direct and subtractively processed dental composite resins by evaluating their water sorption, solubility, surface roughness, and modifications in optical properties after water immersion. This study’s analysis of these characteristics can assist dentists in selecting the optimal material for certain restorations. The expected outcome could help determine if one type of material is better suited for long-term restorations that need more stability. This study examines whether industrially processed CAD/CAM materials demonstrate better performance compared to other options. In clinical practice, where clinicians have to choose between direct and indirect restorations, depending on the clinical scenario, these in vitro findings could guide their decision, potentially leading to more durable and long-lasting outcomes for patients.

Studies investigated the solubility and water sorption characteristics of various restorative materials [30]. However, there are few studies in the same research comparing the water sorption and solubility of direct resin composites in contrast with subtractively produced materials, which is the novelty of this study.

The first null hypothesis was that there is no connection between the water immersion, solubility, the type of monomers in the organic matrix, and the type and percentage of fillers. The second null hypothesis was that immersion in water has no effect on optical properties. The third null hypothesis was that there is no connection between immersion in water and modifications of surface roughness.

## 2. Materials and Methods

### 2.1. Specimen Preparation

This study analyzed three dental direct composite resins and four CAD/CAM materials. Direct composite samples were prepared using a 3D-printed mold, and each resin was applied, compacted, and photopolymerized. CAD-CAM samples were sectioned from CAD/CAM material blocks using an isoparallelograph and processed to achieve final dimensions of 14 × 10 × 1.5 mm. Surface finishing was performed using abrasive paper and polished with Compo+ paste. We used G*Power for the power analysis, where we set an expected effect size (f = 0.25 for a medium effect), a power of 0.80, and an alpha level of 0.05. The number of 10 samples per group met the requirement of 0.80 power for detecting significant effects. Accordingly, seventy samples (10 from each material) were prepared and stored in individual glass containers. Manufacturers and compositions are listed in Table 1 and Table 2.

### 2.2. Water Sorption and Solubility

Water sorption and solubility were evaluated according to ISO 4049:2000 [31]. The samples were cleaned in an ultrasonic bath for 5 min.

All samples were placed in glass containers with silica gel and dehydrated in a desiccator at 37 °C (+/−1 °C). After 22 h, they were stored at 23 °C for 2 h in order to accomplish the 24 h cycle and then weighed using an analytical balance (Kern ABT 100–5 NM, KERN & SOHN GmbH, Balingen, Germany) with a precision of 0.00001 g.

The dehydration and weighing cycles were repeated until a constant mass (m1) was achieved (mass loss < 0.1 mg within 24 h, over 2–3 weeks). The volume was calculated, and the specimens were immersed in 10 mL of distilled water for the following reference days: 1, 7, 14, and 30, and weighed on those days to obtain m2-day 1, m2-day 7, m2-day 14, and m2-day 30. The samples were manipulated using forceps, dried on absorbent paper for 15 s, weighed after 1 min, and then re-immersed in the testing medium.

Afterward, the specimens were reconditioned in desiccators to obtain a final constant mass (m3).

Water sorption (“Wsp”) and water solubility (“Wsl”) were quantified in µg/mm^3^ using Equations (1) and (2).

(1) $$Wsp=\frac{m2-m3}{V}$$

(2) $$Wsl=\frac{m1-m3}{V}$$

where:

m1 is the mass of the conditioned specimen, in µg;

m2 is the mass of the specimen, in µg, after immersion in water for 1, 7, 14, and 30 days;

m3 is the mass of the reconditioned specimen, in µg;

V is the volume of the specimen, in mm^3^.

### 2.3. Surface Roughness Measurements

Surface roughness (Ra, Rz) was measured using a contact 2 µm stylus profilometer (Surftest SJ-201, Mitutoyo, Kawasaki, Japan) with a sampling length of 0.8 mm and an applied force of 0.7 mN. The arithmetic average roughness (Ra) and maximum absolute vertical roughness (Rz) were measured for samples at all stages for each group, material, and stage. These were taken in five different directions, and the average of these five readings was calculated for each surface. Measurements were conducted in three states:

- Initial (Ra0/Rz0): Post-desiccation, after dehydration and obtaining a constant mass (m1).
- Post-immersion (Ra1/Rz1): After 30 days of immersion in distilled water (m2).
- Post-reconditioning (Ra2/Rz2): After re-desiccation, following reconditioning in desiccators to achieve a final constant mass (m3).

### 2.4. Optical and Color Change Measurements

Under D65 standard illumination, the optical CIE Lab* coordinates were determined at each stage with a spectrophotometer (Easyshade IV, Vita Zahnfabrik, Bad Säckingen, Germany).

Before every measurement, the instrument was calibrated; the probe tip was kept at a 90-degree angle to the sample surface.

For each sample, measurements were taken at five different points on the surface, and the average value was calculated on both black (b) and white (w) backgrounds. The WhiBal G7 gray card (White Balance Pocket Card) was used to set the neutral white and black areas.

To evaluate color variances across several criteria, the L*, a*, and b* values were measured. The L* coordinate measures brightness; L* = 0 represents absolute black, and L* = 100 denotes absolute white. Whereas the b* coordinate indicates chromaticity along the yellow (positive value) to blue (negative value), the a* coordinate indicates chromaticity along the red (positive value) to green (negative value).

Translucency (TP), opalescence (OP), and contrast ratio for opacity (CR) were calculated using the following Formulas (3)–(5).

TP = [(Lb − Lw)^2^ + (ab − aw)^2^ + (bb − bw)^2^]^1/2^ (3)

TP values range from 0 (completely opaque) to 100 (completely transparent).

OP = [(ab − aw)^2^ + (bb − bw)^2^]^1/2^ (4)

CR values range from 0 (completely transparent) to 1 (completely opaque).

CR = Yb/Yw = [(L* + 16)/116]^3^ × 100 (5)

The total color change (ΔE*) was calculated using Formula (6):

ΔE* = [(ΔL*)^2^ + (Δa*)^2^ + (Δb*)^2^]^1/2^ (6)

The color change was then converted to NBS units using the following Equation (7):

NBS = ΔE* × 0.92(7)

According to the NBS scale, color changes are categorized as follows: extremely slight (0.0–0.5), slight (0.5–1.5), perceivable (1.5–3.0), marked (3.0–6.0), extremely marked (6.0–12.0), and change to another color (>12.0) (Table 3) [32].

### 2.5. Statistical Analysis

Statistical analysis was performed using Analyse-it software (Analyse-it Software, 6.15.4, Ltd., Leeds, UK). Using the Shapiro–Wilk test for distribution, we determined that the data were generally normally distributed (*p* > 0.05). To make sure the variances were similar across groups, we also ran Levene’s test, which showed no significant differences (*p* > 0.05). Based on these results, using parametric tests was appropriate. Differences between variables were assessed, and comparisons of means were conducted using the unpaired, paired Student’s *t*-test and one-way ANOVA. Statistical significance was set at *p* < 0.05. The Pearson correlation was utilized to analyze the relationship between microroughness and color change and between color parameters. The strength of the association was classified as follows: 0–0.2 (“very weak”), 0.2–0.4 (“weak”), 0.4–0.6 (“moderate”), 0.6–0.8 (“strong”), and 0.8–1.0 (“very strong”). There were negative and positive correlations.

## 3. Results

### 3.1. Water Sorption and Solubility

**Figure 1:**
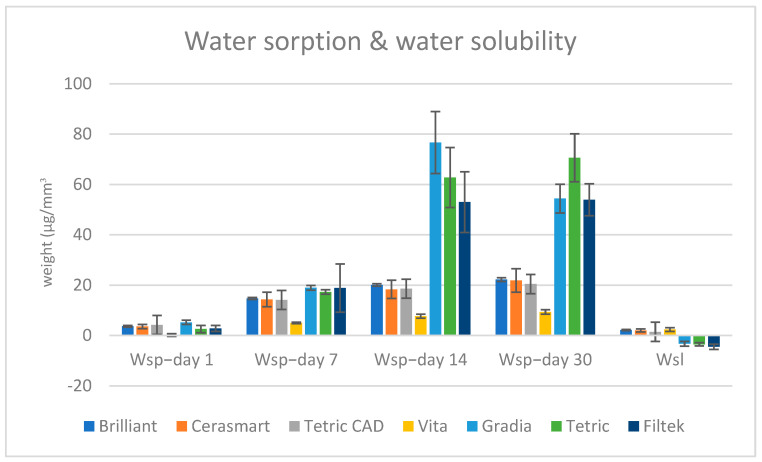
Water sorption and water solubility values of the samples on days 1, 7, 14, and 30 of water immersion.

Table 4 and Figure 1 present the mean values for water sorption (Wsp) and solubility (Wsl) of the seven materials tested after 1, 7, 14, and 30 days of immersion in distilled water.

After one day of immersion in distilled water, Gradia recorded the highest absorption rate (5.19), followed by Tetric CAD (4.19) and Brilliant (3.65). The mean water absorption on day 1 occurred in the following descending order: G > TC > B > C > F > T > V, with the lowest value registered by Vita Enamic.

On day 7, the values showed a significant increase in the following order: G > F > T > B > C > TC > V.

On day 14 of immersion, Gradia recorded the highest absorption rate (76.67), followed by Tetric (62.77), Filtek (52.98), Brilliant (20.09), Tetric CAD (18.59), Cerasmart (18.3), and Vita (7.65): G > T > F > B > TC > C > V.

The order changed after 30 days of immersion: T > G > F > B > C > TC > V.

Following 30 days of immersion, solubility values varied; some materials showed limited mass loss (positive values), while others appeared to have gained weight (negative values), most likely from water absorption. This indicates that the general mass change depends on the balance between water sorption and material dissolution.

The differences in water sorption of the materials studied might be explained by their resin matrix composition and filler quantity. Materials having more hydrophilic monomers like TEGDMA and UDMA, as seen in Tetric and Gradia, tend to absorb more water. Materials using nanoparticle fillers, such as Filtek Z550 and Brilliant, also showed more water absorption, probably because of the increased surface area of the fillers.

The unpaired Student’s *t*-test was used to compare the water sorption of the materials for the reference days 1, 7, 14, and 30, and it showed significant differences (*p* < 0.05) on day one between G and T, G and F, G and V, G and C, G and B, G and TC, T and V, F and V, F and B, F and TC, V and B, V and C, V and TC, and B and TC.

Significant differences (*p* < 0.05) on day seven were found between G and V, G and C, G and B, G and TC, T and V, F and V, V and B, V and C, V and TC, and B and TC.

Significant differences on day 14 (*p* < 0.05) were observed for all pairwise comparisons of the materials, except between T and F, C and B, and C and TC (*p* > 0.05).

Also, statistically significant differences were identified among all pairs of materials on day 30, with the exception of G and F, C and B, and C and TC.

The paired Student’s *t*-test was performed to compare water absorption on reference days 7, 14, and 30 with day 1 of absorption for the same material, with significant differences (*p* < 0.05) being reported among all seven materials.

### 3.2. Optical and Color Changes Measurements

#### 3.2.1. Translucency Parameter

**Figure 2:**
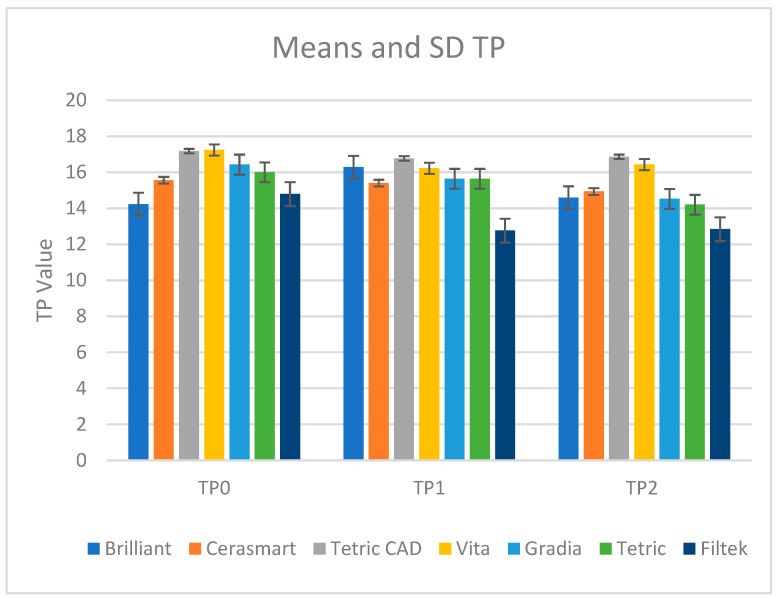
Mean values and standard deviations for the translucency parameter in the desiccated stage (TP0), after 30 days of water immersion (TP1), and in the re-desiccated stage (TP2).

The values for the translucency parameter are presented in Figure 2.

Vita (17.24) recorded the highest TP value in the dehydrated stage (TP0); Brilliant (14.23) recorded the lowest. For TP0, the descending order of values was Vita > Tetric CAD > Gradia > Tetric > Cerasmart > Filtek > Brilliant.

Following 30 days of water immersion (TP1), Tetric CAD had the highest (16.77) value, and Filtek displayed the lowest (12.76). TP1’s order was Tetric CAD > Brilliant > Vita > Gradia > Tetric > Cerasmart > Filtek.

In the re-desiccated stage (TP2), Tetric CAD again recorded the highest value (16.86), while Filtek remained the lowest (12.84). Finally, for TP2, the descending order was Tetric CAD > Vita > Cerasmart > Brilliant > Gradia > Tetric > Filtek.

After water immersion and re-desiccation, Tetric CAD often showed the highest TP values; Filtek regularly recorded the lowest values.

The paired *t*-test results show that between TP0 and TP1, significant changes in translucency were observed for Filtek (*p* = 0.0007) and Brilliant (*p* = 0.002), while other materials showed no significant differences. Between TP0 and TP1, no significant differences were observed for Gradia (*p* = 0.08), Tetric (*p* = 0.42), Cerasmart (*p* = 0.75), Tetric CAD (*p* = 0.69), or Vita (*p* = 0.07), indicating that water immersion did not significantly alter their translucency (Table 5).

Between TP0 and TP2, notable modifications were observed for Gradia (*p* = 0.0005), Tetric (*p* = 0.0008), and Filtek (*p* < 0.0001), whereas Brilliant, Cerasmart, Tetric CAD, and Vita remained unchanged (Table 5).

Filtek showed the most consistent changes across both stages, while other materials demonstrated more stability. This result could be explained by the fact that its filler composition, consisting of zirconia and silica nanoparticle fillers, might facilitate interactions with water. The presence of hydrophilic monomers may increase its susceptibility to water and environmental conditions.

#### 3.2.2. Opalescence Parameter

**Figure 3:**
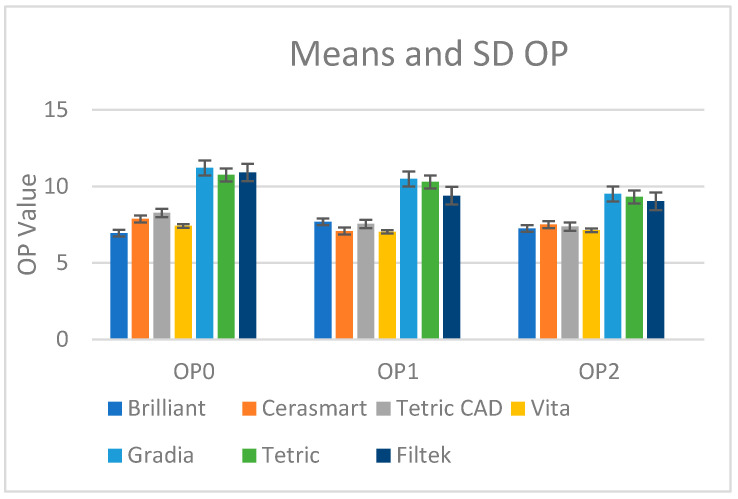
Mean values and standard deviations for the translucency parameter in the desiccated stage (OP0), after 30 days of water immersion (OP1), and in the re-desiccated stage (OP2).

The values for the opalescence parameter are presented in Figure 3.

Dehydration (OP0) resulted in the highest opalescence values for most materials. Gradia had the highest opalescence (11.21), followed by Tetric (10.75), Filtek (10.91), Tetric CAD (8.27), Cerasmart (7.87), Vita (7.42), and Brilliant (6.94). The order for OP0 is Gradia > Filtek > Tetric > Tetric CAD > Cerasmart > Vita > Brilliant.

After water immersion for 30 days (OP1), opalescence values generally decreased compared to OP0. Gradia remained the highest (10.49), followed by Tetric (10.29), Filtek (9.39), Brilliant (7.69), Tetric CAD (7.54), Cerasmart (7.08), and Vita (7.03). The order for OP1 is Gradia > Tetric > Filtek > Brilliant > Tetric CAD > Cerasmart > Vita.

Gradia still had the highest opalescence (9.51) in the re-drying stage, followed by Tetric (9.31), Filtek (9.03), Cerasmart (7.51), Tetric CAD (7.37), Brilliant (7.24), and Vita (7.13). The order for OP2 is Gradia > Tetric > Filtek > Cerasmart > Tetric CAD > Brilliant > Vita.

Dehydration (OP0) revealed the highest opalescence values among all materials; water immersion (OP1) significantly reduced opalescence. Re-drying (OP2) produced some opalescence partial recovery but still below OP0. Gradia regularly displayed the highest opalescence values; Tetric and Filtek followed; Brilliant and Vita had the lowest values across all conditions.

The paired *t*-test results show that regarding the comparison between OP0 and OP1, Gradia shows a significant difference with a *p*-value of 0.0332 (Table 6). Tetric, Filtek, and Brilliant have *p*-values of 0.1527, 0.0918, and 0.1375, respectively, indicating no significant differences. In contrast, Cerasmart and Tetric CAD display highly significant differences, with *p*-values of 0.00001 and 0, respectively. Vita also shows a significant difference with a *p*-value of 0.0331.

For the comparison between OP1 and OP2, Gradia has a *p*-value of 0.0034, indicating strong significance. Tetric demonstrates the highest level of significance with a *p*-value of <0.0001. Filtek and Vita also show significant differences, with *p*-values of 0.0471 and 0.0442, respectively. Cerasmart and Tetric CAD exhibit significant differences as well, with *p*-values of 0.0183 and 0.0121. However, Brilliant has a *p*-value of 0.1115, suggesting no significant difference in this comparison.

Overall, the data highlight the fact that materials like Cerasmart, Tetric CAD, and Gradia (which contain more hydrophilic monomers, such as TEGDMA and UDMA) show consistent statistical significance in both comparisons, while others like Brilliant display less pronounced differences. In conclusion, opalescence parameters of materials like Gradia, Tetric CAD, and Cerasmart were more affected by the hydration and dehydration cycles, while Brilliant, Tetric, Filtek, and Vita demonstrated greater stability.

#### 3.2.3. Contrast Ratio Parameter

**Figure 4:**
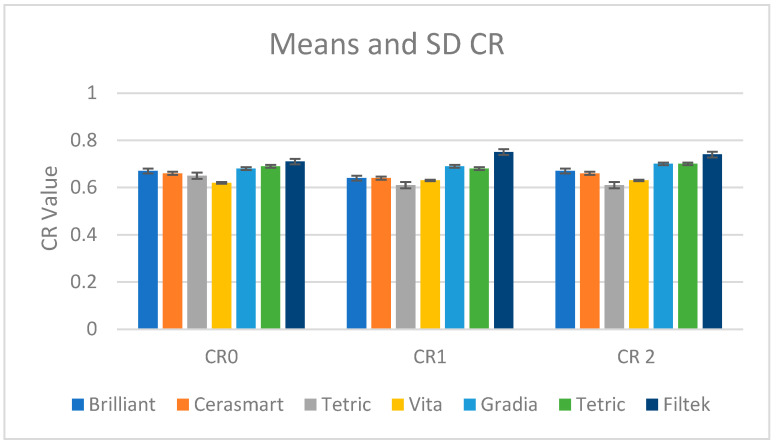
Mean values and standard deviations for the contrast ratio parameter in the desiccated stage (CR0), after 30 days of water immersion (CR1), and in the re-desiccated stage (CR2).

The values for the contrast ratio parameter are presented in Figure 4.

For most materials, the contrast ratio remains relatively stable across the three conditions, indicating minimal changes due to hydration and dehydration cycles.

Brilliant and Cerasmart maintain their CR values consistently, showing minor changes between the stages (0.67 > 0.64 > 0.67 for Brilliant and 0.66 > 0.64 > 0.66 for Cerasmart).

Tetric shows a slight decrease in CR after immersion (0.65 > 0.61 > 0.61), indicating a small reduction in opacity that remains after re-drying.

Vita exhibits stable values throughout the process (0.62 > 0.63 > 0.63), suggesting its contrast ratio is unaffected by hydration or dehydration.

Gradia demonstrates a slight increase in CR (from 0.68 at CR0 to 0.70 at CR2), indicating an increase in opacity after re-drying.

Filtek shows the most noticeable change, with an increase in CR after immersion (0.71 > 0.75 > 0.74), suggesting it becomes slightly more opaque after water absorption but stabilizes slightly after re-drying.

Contrast ratio is generally stable across hydration and dehydration cycles, with small variations for certain materials. Filtek shows the greatest sensitivity, with an increase in opacity after immersion, while materials like Vita, Brilliant, and Cerasmart remain largely unaffected. Gradia and Filtek exhibit slight increases in contrast ratio over time, while Tetric shows a slight reduction in opacity after immersion.

The paired *t*-test results (Table 7) show that between CR0 and CR1, significant changes in contrast ratio were observed for Gradia (*p* < 0.0001) and Tetric (*p* < 0.0001), while other materials showed no significant differences. Gradia and Tetric exhibited large drops in contrast ratio, indicating that dehydration significantly altered their optical properties.

Between CR0 and CR2, significant changes were observed for Gradia (*p* = 0.001), Tetric (*p* = 0.003), and Cerasmart (*p* = 0.0003), with contrast ratios showing considerable variation, particularly after re-dehydration. This suggests that these materials were notably affected by both dehydration and rehydration.

Other materials, including Filtek (*p* = 0.67), Brilliant (*p* = 0.12), Tetric CAD (*p* = 0.29), and Vita (*p* = 0.15), showed no significant changes in contrast ratio between CR0 and CR1 or CR0 and CR2, indicating that water immersion and re-dehydration did not significantly affect their optical properties.

Filtek displayed the most stability across both stages, with minimal changes in contrast ratio, while Gradia, Tetric CAD, and Tetric exhibited more significant fluctuations.

**Figure 5:**
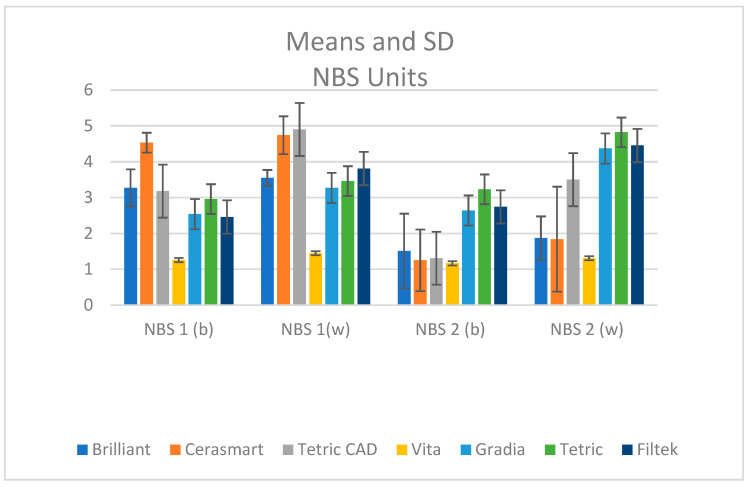
Means and SD for color change expressed in NBS units after 30 days of water immersion (NBS 1) and re-desiccation (NBS 2) on black (b) and white (w) backgrounds.

The changes of color illustrated by NBS Units (Figure 5) were “marked change”, “perceivable”, and “slight change”.

### 3.3. Surface Roughness Measurements

**Figure 6:**
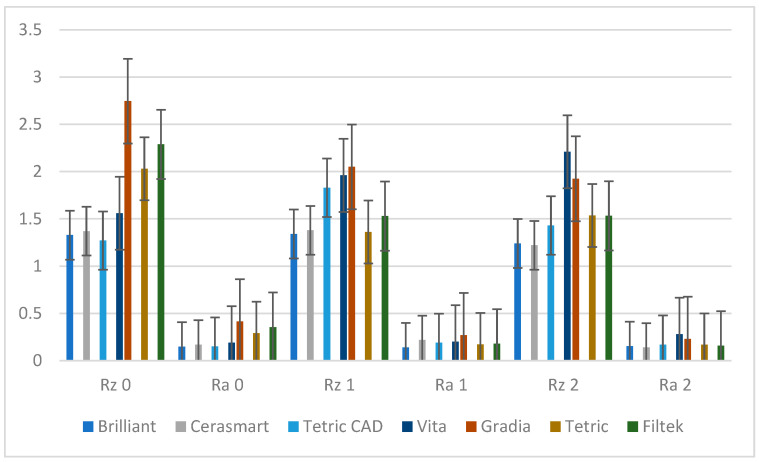
Means and SD corresponding to Rz 0, Ra 0, Rz 1, Ra 1, Rz 2, and Ra 2.

The average values for Rz and Ra, with the corresponding Ra0 (desiccation stage), Ra1 (after 30 days of water immersion), and Ra2 (after re-desiccation), are shown in Figure 6.

The average Ra values exceeded 0.2 μm (clinically acceptable) in the dehydrated stage (Gradia, Filtek, Tetric) but fell below this threshold for Filtek and Tetric after immersion in distilled water and after re-desiccation. Gradia maintained values above the clinically acceptable threshold throughout this study. CAD-CAM maintained all the values within the clinically acceptable limit (exception: Cerasmart Ra1-0.22, Vita Enamic-Ra 2-0.28).

The paired *t*-test revealed significant differences between Ra0-Ra2 for Gradia, Tetric, and Filtek and between Ra0-Ra1 for Gradia, Tetric, and Filtek (Table 8).

Gradia, Tetric, and Filtek exhibited significant increases in roughness (Ra0 → Ra2, Ra0-Ra1) after hydration and re-desiccation (all *p* < 0.05).

Brilliant, Cerasmart, and Vita maintained stable roughness values, indicating resilience to water immersion and desiccation (all *p* > 0.05).

These results may be attributed to both the filler type and monomer composition, monomers with hydrophilic nature influencing the water uptake, which in turn affects surface roughness. CAD-CAM materials with higher filler content and lower water sorption showed more stable surface roughness.

## 4. Discussion

Predicting the long-term performance of composites in the mouth requires an understanding of how they absorb water. The material may undergo dimensional changes and become more susceptible to secondary caries as a result of high sorption. Composites’ mechanical qualities deteriorate with time, and they may release substances that cause allergic responses [15].

Since direct resin composite materials and subtractively produced CAD-CAM materials used for dental restorations are frequently exposed to wet environments, investigating the impact of water sorption on their properties is essential.

Several studies indicate that water absorption in restorative materials weakens the chemical structures and is associated with surface smoothing of the materials [17].

Water sorption and solubility in composite materials are the subjects of multiple investigations. By defining maximum acceptable values for water sorption and solubility in resin-based materials, such as composites and different types of cement, ISO standard 4049 [31] emphasizes the importance of composite–water interactions. Resin-based materials must demonstrate water sorption values of 40 micrograms/mm^3^ or fewer, and for specimens with a diameter of 15 mm and a thickness of 1 mm, solubility values of 7.5 micrograms/mm^3^ or less, to satisfy this requirement [16].

On days 1 and 7, materials satisfied these requirements; on day 30, they did not, and no solubility was noted. Gradia showed the highest sorption rates on reference days 1, 7, and 14, while Tetric had the highest after 30 days.

Other research on the water sorption properties of resin composites indicates that the particular composition of the composite, the kind and amount of fillers used, and the storage environment may affect the degree of water absorption. These studies indicate that the interaction of the polymer matrix with the fillers as well as the storage environment significantly affects the total water sorption of the composite [14].

The type of filler, particle sizes, degree of polymerization, and content of the polymer matrix affect solubility and absorption. Studies have shown that ethoxylated Bis-GMA (Bis-EMA) resins, which lack both the hydroxyl groups of Bis-GMA and the urethane linkages of UDMA, have the lowest water absorption, whereas Bis-GMA-based resins had better water sorption than urethane resins [19,33]. Furthermore, since TEGDMA introduces hydrophilic ether linkages, Bis-GMA resins with higher TEGDMA levels exhibit greater water absorption [34].

In our study, materials were chosen depending on their chemical composition, morphology, and particle size, as filler content and matrix composition vary and could serve to explain the variations in results. Indirect restorations utilize milled CAD/CAM materials (Tetric CAD, Vita Enamic, Brilliant Crios, and Cerasmart), and their clinical indications differ from resin composites (Gradia, Tetric EvoCeram, and Filtek Z550) used for chairside restorations.

We selected these materials because knowing their variations in water sorption, solubility, and surface changes is critical for selecting the right material in a variety of clinical situations. Furthermore, our research aimed to investigate if industrial polymerization of CAD-CAM materials improves performance as compared to direct resin composites.

The absorption of water leads to the expansion of composite resins, resulting in irreversible deterioration. Research indicates that water absorption is reduced in hybrid composites compared to microfiller composites due to the finer particle size. Consequently, considering these aspects, hybrid composites have been frequently utilized in research, and our study also concentrated on them [35].

After 14 and 30 days, respectively, Gradia and Filtek exhibit remarkably high water sorption in this research, peaking at 76.67 and 52.98 on day 14. Given that hydrophilic monomers like UDMA and TEGDMA are known to absorb more water, the high values of water sorption could be related to the chemical composition of these. Gradia also uses Bis-EMA, which has moderate hydrophobicity, but the overall effect is dominated by other hydrophilic components. Zirconia and silica nanoparticles in Filtek could increase surface area, facilitating water sorption.

Tetric and Brilliant also show significant water sorption, with Wsp values rising continuously, especially for Tetric (reaching the highest value—70.6 by day 30). Higher water absorption could be facilitated by the presence of hydrophilic components like TEGDMA in Tetric’s matrix (Bis-GMA and TEGDMA). Brilliant contains tiny silica particles that may interact with water more easily.

Replacing TEGDMA with UDMA or Bis-EMA, as shown in another research, decreases water sorption. High filler content reduces water absorption by minimizing the resin matrix volume. However, nanoparticles, such as those in Filtek Z550, can enhance water interaction due to their large surface area. These outcomes are influenced by both filler type and matrix composition [33].

At only 9.32 by day 30, Vita has the lowest water sorption values of any material. With 86% feldspar and aluminum oxide, Vita Enamic is a hybrid ceramic that is far less susceptible to water sorption than polymer matrices.

The ability of a resin matrix to absorb water depends largely on the filler volume percentage relative to the restoration’s total volume. Materials with lower filler content and a higher resin matrix proportion generally exhibit increased water absorption [36]. In this study, given Gradia’s lowest inorganic filler, this can help to explain the great water sorption attributed to it.

This statement was supported by another investigation on the impact of filler content on the resin structure, which demonstrated that the water absorption values of composite resins with a high percentage of filler content were notably lower than those of resins with a low filler content [37].

Resin composites with quartz fillers were less inclined to water sorption, according to another research, compared to those with zirconia, barium, or zinc glass fillers [38,39].

Our findings that Cerasmart, Tetric CAD, Brilliant Crios, and Gradia perform worse than Vita Enamic are supported by this fact, since barium glass is used as a filler in these products.

The release of unpolymerized monomers upon curing affects the solubility of resin composite materials. Because a larger density of double bonds (C=C) reduces the number of unreacted monomers escaping, a higher degree of monomer conversion results in less dissolution. Furthermore, the dissolving behavior of the material can also be influenced by characteristics such as the bonding agent used, the size of the filler particles, their concentration, and their total surface area [40].

ISO recommendations state that the solubility of the investigated materials could be influenced by the cycles of dehydration of the samples [6]. Consequently, desiccation may not completely remove the absorbed water, and some of it might interact with the resin matrix [6]. This theory helps to explain the negative solubility values of Gradia, Tetric, and Filtek identified in our work, which would represent material water retention during extended immersion. By contrast, Brilliant, Cerasmart, Tetric CAD, and Vita exhibit positive solubility values; Vita has the highest at 2.34.

Vita’s rigid ceramic structure and hydrophilic monomers (UDMA, TEGDMA) may cause matrix deterioration or unreacted component release. Brilliant and Cerasmart’s inorganic fillers stabilize the matrix, reducing solubility. Because Vita Enamic is a hybrid ceramic with a high inorganic content (86% feldspar ceramic enhanced with aluminum oxide), its water sorption and solubility are noticeably reduced. Studies on hybrid ceramics reveal that dense ceramic networks limit water sorption. However, hydrophilic monomers like TEGDMA may increase solubility, according to one study [33].

Although multiple research studies have been conducted on water sorption, differing time intervals, diverse expression units, and varying sample sizes complicate the comparison of raw data [41,42].

The first null hypothesis, which stated that there was no connection between the water immersion, the type of monomers in the organic matrix, and the type and percentage of fillers, was rejected.

The clinical impact of water sorption on dental restorations includes noticeable esthetic deterioration due to discoloration, volumetric changes, and the formation of fissures along the margins. Over time, these effects weaken the restoration and contribute to its eventual failure [43].

A study suggests that how well dental restorations maintain their color over time is strongly influenced by water absorption. This, in turn, depends on how hydrophobic the matrix is and how strong the bond is between the silane and the filler [19].

Among the most frequent causes for replacing dental restorations are unacceptable color changes—whether caused by water absorption and internal deterioration or staining from food pigments. These factors are simulated in in vitro investigations, with clinically defined ΔE thresholds, in order to determine whether the noted changes fit within the limits of acceptable patient perception. Research indicates that values of ΔE < 1 are considered not noticeable by the human eye, so patient perception of color change would be implied in the case of values > 3.3 [44].

Concerning color change, quantified in NBS Units, significant changes (3.0–6.0) and perceptible changes (1.5–3.0) were recorded. In this study, color changes were categorized as “marked”, “perceivable”, or “slight”, with Gradia and Filtek showing higher levels of color change compared to other materials. According to reviews, ΔE levels above 3.3 are considered clinically inappropriate and noticeable to those without specialist training. Therefore, color variations with ΔE values greater than 3.3 are considered unsuitable for clinical applications [44]. In a clinical context, a restoration with a color change (ΔE > 3.3) would imply replacement, especially in the anterior area, which is deemed to have high esthetic demands.

Many studies have highlighted a significant relationship between the long-term color stability of composite materials and a variety of internal and external factors [19].

The discoloration of the resin composite may be significantly influenced by the kind of resin matrix used. Studies have reported that resin composites containing TEGDMA monomers are more susceptible to color change, while Bis-GMA-based resins show higher water sorption [45]. These observations were also found in our study and demonstrated by the poorer performance of direct resin composites, containing monomers such as Bis-GMA and TEGMA, in terms of optical properties.

One study emphasized that the surface smoothness and vulnerability to color change are strongly influenced by the composition of the resin matrix and properties of the filler particles [44]. It has been demonstrated that a lower inorganic filler content in composite resins is associated with more pronounced color change, as the larger resin matrix volume permits greater water sorption [46]. In our study, this statement was demonstrated by the fact that, overall, CAD-CAM materials with a higher inorganic filler percentage showed better optical performance.

The CIE L*a*b color space was used to evaluate the parameters translucency (TP), opalescence (OP), and contrast ratio (CR). Translucency, opacity, fluorescence, and opalescence are some of the optical characteristics of esthetic materials that are important in defining how light interacts with them, which in turn affects how they appear and whether they are suitable for dental restorations [47].

The term “translucency” describes a material’s ability to scatter light while permitting its transmission, making it impossible to see clearly through it. It stands for a condition in between total opacity and total transparency [47].

Human tooth translucency ranges from 15 to 19 at 1 mm thickness; restorative materials can reach up to 25. At 1.5 mm thickness, the average TP values in this investigation varied from 12.76 to 17.24. Vita had the highest TP values in the dehydrated form (17.24), which is comparable to the values found in natural enamel. The lowest values were seen in Filtek (12.76) after 30 days of water immersion. This result can be explained by the fact that Filtek, which consists of zirconium/silica nanoparticles, is more susceptible to water sorption than Vita [38,39], which exhibits good stability due to its high ceramic content (86%).

Opacity, the opposite of translucency, is the degree to which a substance prevents light from passing through [47].

Materials with higher translucency (higher TP) have a lower CR, indicating higher transparency. Tetric and Vita CAD, for example, showed low values of CR and high values of TP. Thus, this was demonstrated in this study, with a TP-CR correlation value (r-0.90) indicating a very strong negative correlation, showing an inverse relationship between translucency and contrast ratio. Given that TP measures the material’s translucency and CR measures its opacity, this is expected. By definition, opacity (CR) decreases with increasing translucency, resulting in a significant negative connection.

Filtek has lower TP values because it is more opaque (greater CR).

Opalescence, on the other hand, is an optical phenomenon where a material looks reddish-orange or brownish when transmitted and bluish when reflected. This effect is brought on by light dispersion inside the material’s structure [36]. The opalescence parameters of human teeth can reach 22 [48].

At 1.5 mm thickness, the average OP values in this investigation varied from 6.94 to 11.21.

The correlation test showed that translucency and opalescence have almost no association, as seen by the extremely weak negative correlation between TP and OP (r-0.10); CR and OP have a high positive association, meaning that when opalescence rises, so does the contrast ratio (r-0.72).

Tetric and Vita CAD closely resemble the properties of typical dental tissues (TP, OP, and CR) and show a better resistance to cycles of hydration and dehydration.

Materials such as Filtek and Gradia are more sensitive to hydration cycles by nature, showing significant changes in TP, OP, and CR.

The second null hypothesis, according to which immersion in water has no effect on optical properties, was rejected.

Regarding the surface roughness, the surface reflects less light, altering optical properties and reducing material strength and durability. Mean roughness (Ra) values below 0.2 μm are generally considered clinically acceptable [49].

This study found average Ra values exceeded 0.2 μm in the dehydrated stage (Gradia, Filtek, Tetric) but fell below this threshold for Filtek and Tetric after immersion in distilled water. Gradia maintained values above the clinically acceptable threshold throughout this study. In the case of CAD-CAM materials, Vita exhibited a clinically acceptable value in the re-dehydrated stage.

Roughness for most materials reduces after 30 days of water exposure. This pattern can be observed in Gradia, Tetric, Filtek, and Brilliant, likely due to the water smoothing the surface or causing leaching of filler particles, therefore reducing surface irregularities.

One study has also shown that water absorption can lead to the softening of the resin matrix, resulting in microcrack formation and resin degradation. This process may cause filler–matrix debonding, altering the surface roughness of the material [50].

Cerasmart, Tetric CAD, and Vita showed more roughness (Ra 1), likely from breakdown of components, matrix weakness, or water-induced swelling. Most materials showed decreased roughness upon re-dehydration, which might have been caused by the matrix’s water being removed, therefore compacting and reducing the surface. Brilliant and Vita, on the other hand, showed higher roughness after the re-dehydration stage, due to long-lasting structural changes; Cerasmart and Tetric CAD showed lower values.

Only in the case of the Vita material was a moderately positive correlation demonstrated between ΔR and ΔE (r = 0.55). For Brilliant, the correlation was weakly negative (r = −0.28); for Cerasmart, it was weakly positive (r = 0.38); for Tetric CAD, it was moderately negative (r = −0.53); for Gradia, it was weakly negative (r = −0.23); for material Tetric, it was weakly positive (r = 0.35); and for material Filtek, it was weakly positive (r = 0.38).

The surface roughness of a restoration material is important, as it is known that a rough surface, with a value exceeding 0.2 µm, could promote biofilm adhesion [48]. The correlation between surface roughness and color changes is also evident, as the accumulation of staining agents promoted by a rough surface leads to color changes over time. Clinically, this suggests that materials with greater roughness require more maintenance or polishing to maintain their esthetics. Therefore, clinicians should take into regard the connection between roughness and color change. Consequently, they should choose materials that can preserve their surface characteristics in the oral environment, providing superior long-term esthetic results and thus reducing the need for early restorative replacement.

In addition, there is a strong positive correlation (r-0.6) between Rz and Ra.

Furthermore, certain composites can minimize hydrolytic degradation and its impact on surface roughness by reducing water sorption through the use of hydrophobic monomers such as Bis-EMA and EBPADMA [50].

The material’s reaction to hydrolytic conditions is greatly influenced by the integrity of the filler–matrix interface. When exposed to water, poor adhesion can cause filler particles to separate, increasing surface roughness [51].

Another research discusses how water-induced deterioration can result in microstructural damage that cannot be repaired by re-dehydration, such as matrix cracking or surface erosion [52].

Thus, the third null hypothesis that immersion in water has no effect on the surface roughness was rejected.

This study’s findings show that CAD/CAM materials (Tetric CAD, Vita Enamic, Brilliant Crios, and Cerasmart) outperformed direct resin composites (Gradia Direct, Tetric EvoCeram, and Filtek Z550) in terms of stability, surface quality, and reduced water sorption over the course of the 30-day testing period.

Clinically speaking, our results provide credibility to the claim that CAD/CAM materials are preferable to direct composites in applications requiring greater durability and accuracy, including applications as veneers, inlays, onlays, and extended posterior restorations.

This study’s limitations include the examination of surface characteristics, water sorption, and optical parameters after only a 30-day immersion period. Future research that tracks the material’s behavior over a longer immersion time should be considered. Moreover, as this is an in vitro study, it does not account for oral dynamics such as salivary enzymes, pH fluctuations, or mechanical stresses. Another limitation is represented by the lack of research on possible monomer release, cytotoxic effects, or breakdown by-products that can affect oral health. These factors could be addressed in future studies. Additionally, even though SEM/AFM analysis, microleakage analysis, and thermocycling were not used in this study, these methods could be included in future research. For example, leakage testing could be linked to changes in surface roughness and material integrity. We also suggest evaluating the behavior of other composite types with regard to changes in surface roughness, optical characteristics, mechanical performance, and water sorption. We also recommend using different aging protocols and immersion media.

## 5. Conclusions

Within the limitations of this study, the following conclusions can be drawn:

1. Regarding overall behavior and the material type (direct composite resin versus subtractively processed materials), Vita Enamic performed the best in terms of water sorption, maintaining optical characteristics, and surface roughness.
2. Regarding the water sorption, Tetric achieved the greatest value after 30 days of immersion, whereas Gradia reported the highest sorption rate on reference days 1, 7, and 14. The hydrophilic monomers (Bis-GMA, UDMA, and TEGMA) in their composition may be the cause of this result, as demonstrated in this research. However, it was shown in this study that the percentage of inorganic material influences the performance, since Vita, which had the greatest inorganic content, had the lowest rate of water sorption.
3. In terms of surface roughness, the immersion process influenced the surface roughness, with varying results depending on the material. The clinically acceptable value after immersion was exceeded in the case of Gradia and Cerasmart. The Vita material was the only one to exhibit a positive connection between roughness variation (ΔR) and color change (ΔE), indicating that roughness has a greater impact on color perception in this material.
4. Regarding the optical parameters, materials like Gradia, Tetric, and Filtek showed significant changes in translucency, opalescence, contrast ratio, and surface roughness after hydration and re-desiccation. Brilliant, Cerasmart, Tetric CAD, and Vita demonstrated high stability in optical properties, making them more resistant to hydration effects.
5. The differences in the surface roughness and optical parameters highlight the need to consider a material’s specific behavior in an oral environment, especially with regard to appearance and durability.

## Figures and Tables

**Table 1:** Direct resin composites used in this study.

Material	Manufacturer	Matrix Composition	Filler Type	Filler Content (%)
Gradia Direct Anterior A2	GC Corporation, Tokyo, Japan	UDMA, Bis-EMA, dimethacrylates, and trimethacrylates	Barium glass, silica, and pre-polymerized resin fillers	69
Tetric Evo Ceram A2	Ivoclar Vivadent, Schaan, Liechtenstein	Bis-GMA, Bis-EMA, and TEGDMA	Pre-polymerized fillers and silica	75
Filtek Z550 A2	3M ESPE, Saint Paul, MN, USA	Bis-GMA, UDMA, TEGDMA, BIS-EMA, and PEGDMA	Zirconia/silica nanoparticles	78.5

**Table 2:** CAD CAM materials used in this study.

Material	Type	Manufacturer	Filler	Monomer	Shade/Translucency
Vita Enamic (V)	Hybrid ceramic	VITA Zahnfabrik, Bad Säckingen, Germany	Feldspar ceramic enriched with aluminum oxide 86%	UDMA and TEGDMA	A2/MT
Cerasmart (C)	CAD/CAM composite resin	GC Corportion Tokyo, Japan	Silica and barium glass 71%	UDMA, Bis-MEPP, and DMA	A2/MT
Tetric CAD (T)	Composite resin	Ivoclar Vivadent, Liechtenstein	Nano-filled 70% with barium glass and silicon dioxide	Bis-GMA, Bis-EMA, TEGDMA, and UDMA	A2
Brilliant Crios (B)	Composite resin	Coltene/Whaledent, Alstatten, Switzerland	Amorphous silica particles (<20 nm) and glassy ceramic, barium particles (<1.0 µm)	BisGMA, UDMA, and TEGDMA	A2

**Table 3:** Levels of color change, according to the NBS.

NBS Units	Color Change Description
0.0–0.5	Extremely slight change
0.5–1.5	Slight change
1.5–3.0	Perceivable
3.0–6.0	Marked change
6.0–12.0	Extremely marked change
12.0 or more	Change to another color

**Table 4:** Average values of water sorption and solubility for all seven materials.

	Wsp-Day 1	Wsp-Day 7	Wsp-Day 14	Wsp-Day 30	Wsl
Brilliant	3.65	14.71	20.09	22.19	2.17
Cerasmart	3.54	14.33	18.3	21.89	2
Tetric CAD	4.19	14.12	18.59	20.43	1.43
Vita	0.16	5	7.65	9.32	2.34
Gradia	5.19	18.93	76.67	54.37	−3.28
Tetric	2.59	17.3	62.77	70.6	−3.53
Filtek	2.8	18.84	52.98	53.94	−4.49

**Table 5:** The *p*-value for statistical comparisons between stages TP0-TP1 and TP0-TP2 across materials.

	Gradia	Tetric	Filtek	Brilliant	Cerasmart	Tetric Cad	Vita
TP0-TP1	0.08	0.42	0.0007	0.002005	0.75	0.69	0.07
TP0-TP2	0.0005	0.0008	<0.0001	0.13	0.3	0.76	0.14

**Table 6:** The *p*-value for statistical comparisons between stages OP0-OP1 and OP0-OP2 across materials.

	Gradia	Tetric	Filtek	Brilliant	Cerasmart	Tetric Cad	Vita
OP0-OP1	0.033	0.152	0.091	0.137	0.00001	0.000000	0.033
OP1-OP2	0.003	<0.0001	0.047	0.111	0.018	0.012	0.044

**Table 7:** The *p*-value for statistical comparisons between stages CR0-CR1 and CR0-CR2 across materials.

	Gradia	Tetric	Filtek	Brilliant	Cerasmart	Tetric Cad	Vita
CR0-CR1	0.552	0.596	0.0002	0.137	0.146	0.489	0.127
CR0-CR2	0.087	0.058	0.0004	0.111	0.529	0.507	0.233

**Table 8:** The *p*-values for statistical comparisons between stages Ra0-Ra1 and Ra0-Ra2 across materials.

	Gradia	Tetric	Filtek	Brilliant	Cerasmart	Tetric Cad	Vita
Ra0-Ra1	0.02	0.018	0.019	0.656	0.137	0.182	0.896
Ra0-Ra2	0.004	0.001	0.0004	0.957	0.234	0.516	0.113

## Data Availability

The original contributions presented in this study are included in the article. Further inquiries can be directed to the corresponding author.
